# Early surgery versus conservative treatment in patients with traumatic intracerebral hematoma: a CENTER-TBI study

**DOI:** 10.1007/s00701-023-05797-y

**Published:** 2023-09-25

**Authors:** Inge A. M. van Erp, Thomas A. van Essen, Hester Lingsma, Dana Pisica, Ranjit D. Singh, Jeroen T. J. M. van Dijck, Victor Volovici, Angelos Kolias, Lianne D. Peppel, Majanka Heijenbrok-Kal, Gerard M. Ribbers, David K. Menon, Peter Hutchinson, Bart Depreitere, Ewout W. Steyerberg, Andrew I. R. Maas, Godard C. W. de Ruiter, Wilco C. Peul, the CENTER-TBI Investigators, Participants

**Affiliations:** 1 University Neurosurgical Centre Holland, LUMC, HMC, HAGA, Leiden and The Hague, The Netherlands; 2 Centre for Medical Decision Making, Department of Public Health, Erasmus MC—University Medical Centre, Rotterdam, The Netherlands; 3 Department of Neurosurgery, Erasmus MC—University Medical Centre, Rotterdam, The Netherlands; 4 Division of Neurosurgery, Department of Clinical Neurosciences, University of Cambridge and Addenbrooke’s Hospital, Cambridge, UK; 5 NIHR Global Health Research Group on Neurotrauma, University of Cambridge, Cambridge, UK; 6 Rijndam Rehabilitation and Department of Rehabilitation Medicine, Erasmus MC—University Medical Centre, Rotterdam, The Netherlands; 7 Division of Anaesthesia, Addenbrooke’s Hospital, University of Cambridge, Cambridge, UK; 8 Department of Neurosurgery, University Hospital KU Leuven, Leuven, Belgium; 9 Department of Biomedical Data Sciences, Leiden University Medical Centre and Haaglanden Medical Centre, Leiden and The Hague, The Netherlands; 10 Department of Neurosurgery, Antwerp University Hospital and University of Antwerp, Edegem, Antwerp, Belgium

**Keywords:** Conservative treatment, Contusion, Neurosurgery, Surgical treatment, Traumatic intracerebral hematoma

## Abstract

**Purpose:**

Evidence regarding the effect of surgery in traumatic intracerebral hematoma (t-ICH) is limited and relies on the STITCH(Trauma) trial. This study is aimed at comparing the effectiveness of early surgery to conservative treatment in patients with a t-ICH.

**Methods:**

In a prospective cohort, we included patients with a large t-ICH (< 48 h of injury). Primary outcome was the Glasgow Outcome Scale Extended (GOSE) at 6 months, analyzed with multivariable proportional odds logistic regression. Subgroups included injury severity and isolated vs. non-isolated t-ICH.

**Results:**

A total of 367 patients with a large t-ICH were included, of whom 160 received early surgery and 207 received conservative treatment. Patients receiving early surgery were younger (median age 54 vs. 58 years) and more severely injured (median Glasgow Coma Scale 7 vs. 10) compared to those treated conservatively. In the overall cohort, early surgery was not associated with better functional outcome (adjusted odds ratio (AOR) 1.1, (95% CI, 0.6–1.7)) compared to conservative treatment. Early surgery was associated with better outcome for patients with moderate TBI and isolated t-ICH (AOR 1.5 (95% CI, 1.1–2.0); *P* value for interaction 0.71, and AOR 1.8 (95% CI, 1.3–2.5); *P* value for interaction 0.004). Conversely, in mild TBI and those with a smaller t-ICH (< 33 cc), conservative treatment was associated with better outcome (AOR 0.6 (95% CI, 0.4–0.9); *P* value for interaction 0.71, and AOR 0.8 (95% CI, 0.5–1.0); *P* value for interaction 0.32).

**Conclusions:**

Early surgery in t-ICH might benefit those with moderate TBI and isolated t-ICH, comparable with results of the STITCH(Trauma) trial.

**Supplementary Information:**

The online version contains supplementary material available at 10.1007/s00701-023-05797-y.

## Background

Traumatic brain injury (TBI) is often accompanied by intracranial hemorrhages, consisting of extradural (EDH), subdural (ASDH), and/or intracerebral hematomas (t-ICH). Early surgery in clinically deteriorating patients with EDH or ASDH is generally accepted and recommended in international guidelines [12]. T-ICH is more common than any extra-axial hematomata, and studies have demonstrated that up to 41% of t-ICH patients have an unfavorable outcome [26].

Most patients with t-ICHs do not require surgical intervention because the lesions are small or scattered. However, development of mass effect from larger lesions may result in further neurological deterioration and ultimately, due to rising intracranial pressure (ICP), severe disability, or death [1]. The aim of early surgery in t-ICH is to prevent this secondary brain injury, which can be achieved either through removal of the t-ICH, and/or by decompressive craniectomy (DC), mitigating the extent of ICP rise.

Evidence on the clinical effectiveness of early surgery in t-ICH is limited. It relies mostly on the Surgical Trial in Traumatic Intracerebral Hemorrhage (STITCH) Trauma study [16]. Patients with a t-ICH > 10 mL for whom the treating neurosurgeon was in “clinical equipoise” about the benefits of surgery compared to conservative treatment were randomized. The trial was halted early due to an imbalance in patient recruitment per country. An absolute benefit, although not significant, of 10·5% was observed on the functional outcome at 6 months when performing early surgery. Moreover, significantly more deaths were reported in the conservative treatment group (33% vs. 15%, *P* = 0.006), and the subgroup with a GCS 9–12 showed a trend toward better functional outcome with early surgery (odds ratio 0.5 (95% CI 0.2–1.3)). The sample size of the initial power calculation was not reached, leaving the possibility open of a chance finding. Therefore, the beneficial effect of surgery in t-ICH patients is still a matter of debate [3]. This might partly be caused by the lack of a demonstrable effect as well as by doubts on the generalizability of this randomized trial in a “clinical equipoise” setting toward everyday clinical practice [5, 8, 17]. Results from a controlled, experimental setting might not apply to routine circumstances due to a different, and more heterogeneous patient population, lack of evidence-based guideline adherence, and other uncontrolled factors in daily practice [6, 9, 19]. Studies in unselected populations are important to complement evidence generated from clinical trials. This study is aimed at evaluating the effectiveness of early surgery as compared to conservative treatment among patients with t-ICH in an observational cohort representing clinical practice.

## Methods

### Design

The study protocol for this multicenter prospective observational cohort study was previously published [20]. We report according to the Strengthening The Reporting of Observational Studies in Epidemiology statement [23].

### Patient inclusion

Patients were enrolled in the observational cohorts of the CENTER-TBI and Net-QuRe studies, recruiting patients between 2014 and 2017 and 2015 and 2020, respectively [11]. These databases included patients with TBI and excluded those with severe pre-existing neurological disorders that would confound outcome measurements. Approval by the medical ethics committees of all participating centers was obtained. For this study, inclusion was as follows: (1) large (as judged by the treating neurosurgeon on call) t-ICH on CT-scan within 48 h of the injury, (2) age > 18 years, (3) hospital admission, and (4) complete data on primary endpoint. The exclusion criterion was a moribund prognosis on admission, as the treating neurosurgeon judged these patients either to be brain dead on admission or to have an extremely poor prognosis, rendering treatment futile. Informed written or oral consent by patients or legal representatives was obtained according to local legislation.

### Interventions

The two interventions were early surgery or initial conservative treatment. Interventions were classified based on the treatment decision directly after the CT-scan on which the diagnosis of a large t-ICH was made. This could have been the admission CT-scan or a subsequent CT-scan within 48 h of injury (comparable with the STITCH(Trauma) trial) in case of a developing t-ICH. Surgery consisted of evacuation of the hematoma with a craniotomy, and/or a DC, defined as removal of a large portion of the skull to mitigate ICP increase. DC might have been accompanied by hematoma evacuation and could have been bifrontal or a hemicraniectomy. Evacuation of a t-ICH might have also been accompanied by removal of an EDH or ASDH. Conservative treatment consisted medical management (i.e., sedation, hyperosmolar therapy, and hyperventilation) with or without ICP monitoring or extracranial ventricular drainage for prevention and treatment of intracranial hypertension. Conservative treatment could have been accompanied by a delayed surgery if deemed appropriate at a later phase. Neurosurgeons were asked after each CT-scan if and why surgery was indicated.

### Outcome measurements and endpoints

The primary endpoint was functional outcome assessed with the 6-month Glasgow Outcome Scale Extended (GOSE) score [25]. The GOSE is an ordinal scale ranging from 8 (no symptoms) to 1 (death). Secondary endpoints were in-hospital mortality, hospital length of stay (LOS), and “treatment failure” during the hospital admittance, defined as delayed cranial surgery (> 48 h of injury) for patients in the conservative treatment group and a second surgery in patients in the early surgery group. This could either consist of a craniotomy or DC. Moreover, GOSE was dichotomized at various levels (GOSE 7–8 vs. 1–6, GOSE 5–8 vs. 1–4, and GOSE 4–8 vs. 1–3). Last, quality of life at 6 months was assessed using the Quality of Life after Brain Injury Questionnaire (Qolibri) [24].

### Statistical analysis

Baseline and treatment characteristics are presented using descriptive analysis with standardized mean differences between treatment groups. Baseline prognosis for 6-month mortality and unfavorable outcome is summarized using the International Mission for Prognosis and Analysis of Clinical Trials in TBI score (core model) [10].

The predefined primary outcome analysis had a comparative effectiveness design with instrumental variable (IV) analysis [15, 20]. To quantify and compare the between-center treatment variation that is not explained by case-mix factors or attributable to chance, the median odds ratio (MOR) was calculated. Moreover, comparison in the median t-ICH volume between centers was calculated (ANOVA-test) to determine the between-center variation in hematoma volume operated on. The models with and without random effect for center were compared with the likelihood ratio test to determine the significance of the between-center variation. The acquired cohort did not meet the predefined requirements to allow for IV analysis due to lack of treatment variability and small sample size. Instead, multivariable proportional odds logistic regression with treatment strategy as a binary variable and the 8-point ordinal GOSE as outcome variable was performed, with covariate adjustment for age, baseline GCS and pupil reactivity, hematoma volume and laterality, midline shift, and a concomitant EDH and/or ASDH on the scan used for decision making. The subsequent adjusted common odds ratio (AOR) indicates the odds of a more favorable outcome for patients who received early surgery compared to patients who did not.

Secondary outcomes were analyzed with logistic or linear regression with covariate adjustment, resulting in AORs or betas with corresponding 95% confidence intervals (CI).

A sensitivity analysis using propensity score matching (PSM) was undertaken. The aforementioned confounding variables were included as independent variables in the PSM procedure, which was carried out by balanced parallel (1:1) using a nearest neighbor approach with a caliper of 0.10. A second sensitivity analysis explored the impact of patients with a moribund prognosis on admission. To explore selection bias, we simulated these patients to be in (1) the conservative treatment group with poor outcome (GOSE = 1), (2) the early surgery group with poor outcome (GOSE = 1), (3) the conservative treatment group with good outcome (GOSE = 8), and (4) the early surgery group with good outcome (GOSE = 8). A third sensitivity analysis was performed specifying treatment as a continuous variable “time from admission to surgery.” Time to surgery was defined by time from admission to treatment, including early surgery patients and initial conservative treatment patients who required delayed surgery, therefore independent of treatment group. Finally, an analysis comparing DC versus craniotomy within the early surgery group was performed.

Confirmatory subgroup analyses were performed in predefined subgroups based on: age (< 65 or ≥ 65 years), TBI severity (mild, moderate, and severe; respectively, GCS 13–15, 9–12, and 3–8), isolated t-ICH (without concomitant ASDH or EDH) vs. non isolated, t-ICH volume (using median split ≤ 33 and > 33 cc), timing to development of large t-ICH (acute: large t-ICH on first CT-scan or delayed: small t-ICH on first CT-scan blossoming to large within 48 h), and location of the largest t-ICH (frontal, temporal, occipital, and parietal). Interactions of subgroup analyses were tested using the subgroup-defining variable (variable x intervention) and conservative treatment as reference. Results from subgroup analyses were presented in forest plots. No adjustments for multiple tests were made.

Statistical analyses were performed using R software version 4.0.4 and RStudio version 1.1.463 with add-ons. *P* values less than 0.05 were considered significant. Missing baseline data were multiply imputed with the “mice” package (*n* = 5), assuming data to be missing at random.

## Results

**Fig. 1:**
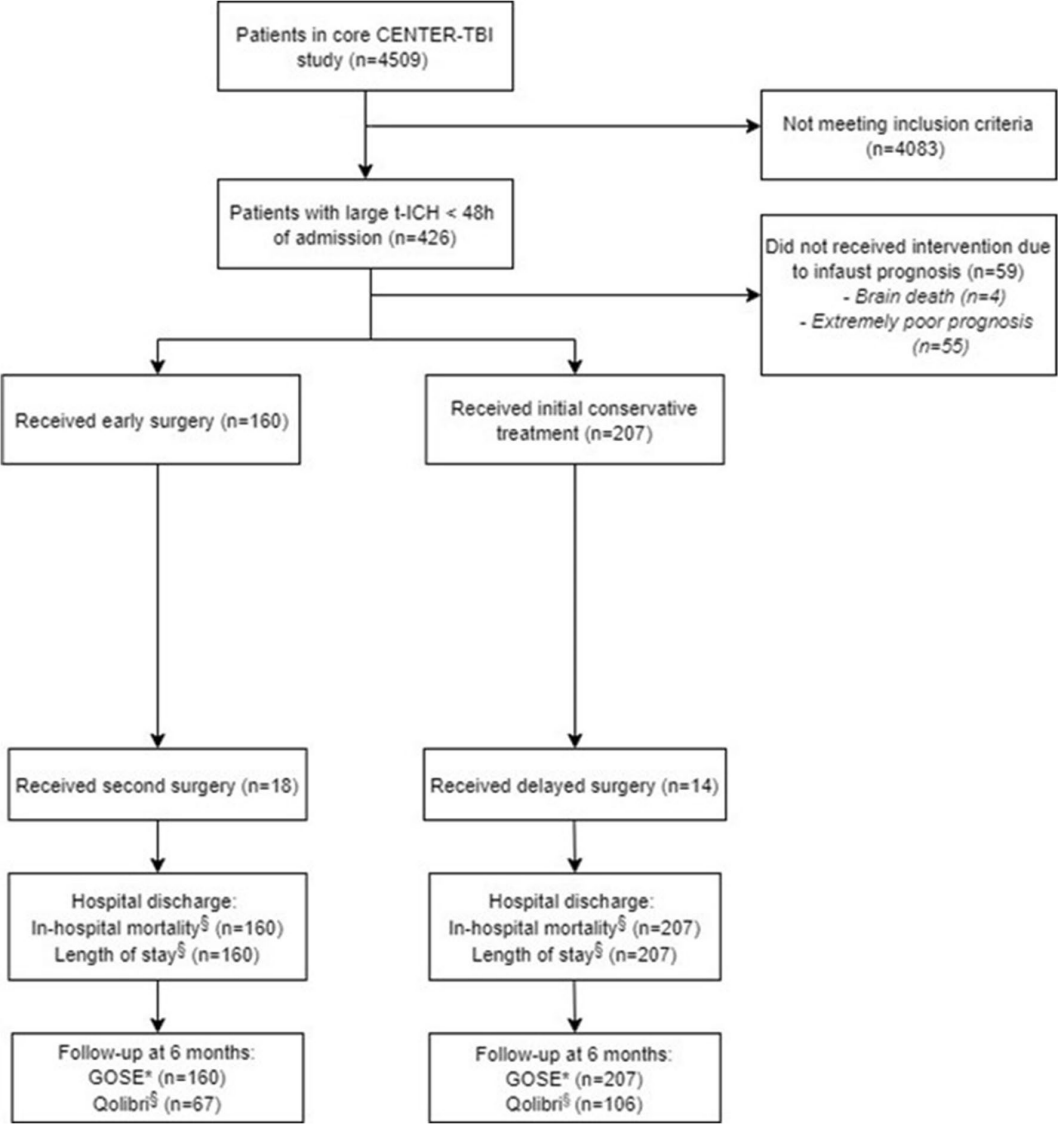
Flow diagram of study population and data analyses. *Primary outcome. ^§^Secondary outcomes. Qolibri exclusion criteria is patients with GOSE 1 (death) or GOSE 2/3 (vegetative state/lower severe disability). Abbreviations: t-ICH, traumatic intracerebral hematoma; GOSE: Glasgow Outcome Scale Extended, Qolibri: Quality of Life after Brain Injury Questionnaire

Overall, 4509 patients were included in the databases, of whom 426 patients met the eligibility criteria. Fifty-nine patients were excluded from primary analysis due to a moribund prognosis on admission (supplemental Table 1). All of the moribund prognosis patients died during admission and none of them received surgery. Of the remaining 367 patients, 207 patients had initial conservative treatment, whereas 160 patients had early surgery (Fig. 1).

Patients in the early surgery group were younger (median 54 vs. 58 years), healthier (ASAPS “healthy” 57% vs. 43%), and more severely injured (median baseline GCS 7 vs. 10) compared to the initial conservative treatment group (Table 1). Comparing radiological features on the last CT-scan prior to surgical decision making, more patients in the early surgery group had a concomitant large EDH (13% vs. 3%), large ASDH (46% vs. 11%), midline shift (70% vs. 42%), and compressed basal cisterns (54% vs. 24%). The volume and location of the largest t-ICH, proportion of patients with two or more t-ICHs, unilaterality of t-ICHs, and the number of anatomic regions involved did not differ between groups. The baseline estimated unfavorable outcome (CRASH-CT score) was 64 [40, 79] in the early surgery group, 55 [43, 75] in the initial conservative treatment group, and 82 [74, 92] in the patients with a moribund prognosis.

**Table 1:** Baseline and radiological characteristics of patients with t-ICH comparing early surgery versus initial conservative treatment

	Early surgery	Initial conservative treatment	SMD	Missing (%)
*n*	160	207		
Age (median (IQR))	54 (35, 64)	58 (42, 71)	0.3	0
Male (%)	122 (76)	147 (71)	0.1	0
Cause of injury (%)			0.3	7
Road traffic incident	48 (30)	65 (31)		
Incidental fall	75 (47)	112 (54)		
Other non-intentional injury	7 (4)	6 (3)		
Assault/violence	8 (5)	8 (4)		
Suicide attempt	4 (3)	2 (1)		
Other	2 (1)	5 (2)		
ASAPS (%)			0.4	6
Healthy	91 (57)	89 (43)		
Mild systemic disease	40 (25)	81 (39)		
Severe systemic disease	15 (9)	28 (14)		
Threat to life	1 (1)	1 (1)		
Antithrombotic medication (%)			0.3	7
No	128 (80)	153 (74)		
Yes, anticoagulants	4 (3)	16 (8)		
Yes, platelet aggregation inhibitors	13 (8)	23 (11)		
Yes, both	1 (1)	2 (1)		
Hypoxia (%)^§^			0.2	8
No	123 (77)	176 (85)		
Definite	17 (11)	10 (5)		
Suspect	6 (4)	6 (3)		
Hypotension (%)^¥^			0.3	8
No	124 (78)	179 (87)		
Definite	14 (9)	8 (4)		
Suspect	6 (4)	5 (3)		
GCS (median (IQR))	7 (3, 13)	10 (6, 14)	0.4	5
GCS motor (median (IQR))	4 (1, 6)	5 (1, 6)	0.3	3
Pupil reactivity (%)			0.2	7
Both reacting	113 (76)	160 (83)		
One reacting	11 (7)	13 (7)		
Both unreacting	25 (17)	19 (10)		
ISS (median (IQR))	27 (25, 39)	26 (18, 38)	0.3	0
AIS head (median (IQR()	5 (5, 5)	5 (4, 5)	0.6	0
TBI severity (%)^∞^			0.4	5
Mild	40 (27)	66 (33)		
Moderate	23 (15)	51 (26)		
Severe	87 (58)	81 (41)		
Epidural hematoma (%)^α^			0.5	1
No	113 (71)	175 (85)		
Small	21 (13)	26 (13)		
Large	21 (13)	6 (3)		
Acute subdural hematoma (%)^α^			0.9	0
No	37 (23)	68 (33)		
Small	48 (30)	117 (57)		
Large	74 (46)	22 (11)		
Subarachnoid hemorrhage (%)			0.3	0
No	26 (16)	52 (25)		
Basal	18 (11)	16 (8)		
Cortical	88 (55)	100 (48)		
Basal and cortical	28 (18)	39 (19)		
Depressed skull fracture (%)			0.4	0
No	108 (68)	171 (83)		
Closed	35 (22)	29 (14)		
Open	16 (10)	7 (3)		
Diffuse axonal injury (%)			0.1	8
No	119 (75)	162 (78)		
Yes	25 (16)	31 (15)		
Midline shift (%)^λ^	112 (70)	86 (42)	0.6	0
Midline shift, mm (median (IQR))	7 (5, 12)	5 (3, 6)	0.4	50
Compressed basal cisterns (%)	86 (54)	49 (24)	0.7	1
Volume of largest t-ICH (cc) (median (IQR))	29 (12, 51)	28 (14, 44)	0.1	6
Location of largest t-ICH (%)			0.4	6
Frontal	58 (83)	48 (73)		
Temporal	9 (13)	16 (24)		
Occipital	1 (1)	0 (0)		
Parietal	1 (1)	2 (3)		
Two or more t-ICHs (%)	52 (73)	45 (67)	0.1	6
All t-ICHs unilateral (%)	25 (35)	22 (33)	0.1	6
Two or more regions involved (%)	56 (80)	49 (74)	0.5	6
Predicted probability of 6 month mortality (median (IQR))^*^	45 (26, 64)	37 (26, 59)	0.3	38
Predicted probability of 6 month unfavorable outcome (median (IQR))^*^	65 (40, 79)	55 (43, 75)	0.2	38

^§^Definite hypoxia is defined as a documented PaO2 < 8 kPA (60 mmg Hg) and/or SaO2 < 90% in pre-hospital or ER phase. Suspected hypoxia was scored if the patient did not have documented hypoxia by PaO2 or SaO2, but there was a clinical suspicion, as evidenced by for example cyanosis, apnea, or respiratory distress. ^¥^Definite hypotension is defined as a documented systolic BP < 90 mm Hg in pre-hospital or ER phase. Suspected hypotension was scored if the patient did not have a documented low BP, but was reported to be in shock or have an absent brachial pulse (not related to injury of the extremity). ^∞^Classified as mild TBI (GCS 15–13), moderate TBI (GCS 9–12), and severe TBI (GCS < 9). ^α^Small and large as judged by the treating physician. ^λ^Presence of midline shift is classified as being more than 5 mm. ^*^Calculated using the International Mission for Prognosis and Analysis of Clinical Trials in TBI (IMPACT) score (core model). Percentage missing includes those with a GCS > 12. Abbreviations: *AIS*, Abbreviated Injury Scale; *ASAPS*, American Society of Anesthesiologists classification system; *GCS*, Glasgow Coma Scale; *IQR*, interquartile range; *ISS*, Injury Severity Score; *SMD*, standardized mean difference; *t-ICH*, traumatic intracerebral hematoma

In the early surgery group, 18% had (an episode of) neurological worsening after their surgery. After conservative treatment, 14% had neuro-worsening (Table 2). Radiologically, in the early surgery group, 31% of patient had a progression of their t-ICH on CT-scan compared to 14% in the conservatively treated group. More patients (80%) in the early surgery group received an ICP monitor compared to the conservatively treated group (54%). Initial and continuous ICP and cerebral perfusion pressure measurements did not differ between the two treatment groups in patients with implanted monitoring devices. Most surgeries in the early surgery group were performed within three hours of admission. Delayed surgeries performed after a median post-admission time of 5 days in the initial conservative treatment group were mostly DCs. Fifty percent of second surgeries in the early surgery group were craniotomies, the remaining being DCs. The median time from admission to second surgery was 4 days.

**Table 2:** Treatment characteristics and clinical course of patients with t-ICH comparing early surgery versus initial conservative treatment

	Early surgery	Initial conservative treatment	SMD	Missing (%)
*n*	160	207		
Timing from admission to surgery, hours (median (IQR))	3 (1, 10)	N/A	N/A	0
Method of first surgery (%)			N/A	0
Craniotomy	79 (49)	N/A		
Decompressive craniectomy	81 (51)	N/A		
Any neuroworsening (%)^a^	28 (18)	28 (14)	0.1	0
Progression on CT (%)^b^	50 (31)	30 (14)	0.4	21
Treatment failure (%)*	18 (11)	14 (7)	0.2	0
Timing from admission to second or delayed surgery, hours (median (IQR))	84 (38, 202)	111 (83, 130)	0.3	46
Method of second or delayed surgery (%)			0.4	36
Craniotomy	9 (50)	2 (14)		
Decompressive craniectomy	9 (50)	7 (50)		
Extracranial surgery (%)^¥^	38 (24)	39 (19)	0.1	0
ICP (median (IQR))^§^	13 (9, 17)	13 (10, 16)	0.1	37
ICP monitor (%)	128 (80)	112 (54)	0.6	0
ICP device (%)			0.3	35
Ventricular	23 (18)	29 (26)		
Parenchymal	92 (72)	79 (70)		
Other	12 (9)	5 (4)		
CPP (median (IQR))^§^	74 (67, 78)	74 (69, 79)	0.2	37
TIL (median (IQR))^§^	7 (3, 11)	4 (1, 9)	0.4	9
ICU length of stay, days (median (IQR))	14 (6, 22)	12 (5, 21)	0.1	13
Dead in hospital (%)	42 (26)	44 (21)	0.0	0
Death cause (%)			0.0	78
Primary head injury	19 (45)	18 (38)		
Secondary intracranial damage	14 (33)	10 (21)		
Systemic trauma	1 (2)	0 (0)		
Medical complications	3 (7)	11 (23)		
Other	3 (7)	1 (2)		
Discharge destination (%)			0.3	40
Other hospital	26 (27)	41 (33)		
Rehab unit	42 (44)	45 (36)		
Nursing home	6 (6)	4 (3)		
Home	18 (19)	31 (25)		
Other	4 (4)	2 (2)		

^a^Neuroworsening is defined as a spontaneous decrease in the Glasgow Coma Scale motor score ≥ 2 points (compared with the previous examination), a new loss of pupillary reactivity, development of pupillary asymmetry ≥ 2 mm, and/or deterioration in neurological or CT status sufficient to warrant immediate medical or surgical intervention. ^b^Progression on the CT scan during the hospital course is defined as an increase in initial lesion and/or the development of a new lesion. *Treatment failure is defined as patients in the initial conservative treatment group who are operated at a delayed moment or patients in the early surgery group who are operated again. ^¥^Extracranial surgery could include damage control thoracotomy, damage control laparotomy, extraperitoneal pelvic packing, external fixation limb, or cranio-maxillo-facial reconstruction. ^§^Calculated as median over the first seven days after TBI. Abbreviations: *ICP*, intracranial pressure; *CPP*, cerebral perfusion pressure; *IQR*, interquartile range; *TIL*, therapy intensity level; *ICU*, intensive care unit; *SMD*, standardized mean difference

The most common reasons for initial conservative treatment were “no surgical lesion” (29%), “lesion present, but little/no mass effect” (22%), and “lesion present, but acceptable/good neurologic condition” (19%). The main reasons for early surgery were “emergency/lifesaving” (42%), “mass effect on CT” (21%), “clinical deterioration” (7%), and “(suspicion of) raised ICP” (7%) (supplemental Fig. 1a/1b, supplemental Fig. 5a/5b).

The median volumes of t-ICH were comparable between centers (SMD 0.1, *P* value 0.34; supplemental Fig. 3). The proportion of early surgery ranged from 13 to 48% between centers. The MOR for acute surgery is 1.4 (*P* = 0.27) (supplemental Fig. 4). The MOR represents relatively small regional treatment differences, and therefore, center is not strongly associated with treatment strategy.

Both the primary analysis with multivariable regression and the sensitivity analysis using PSM showed no difference in GOSE between early surgery vs. initial conservative treatment (AOR 1.1 (95% CI, 0.6–1.7) and AOR 1.1 (95% CI, 0.8–1.5), respectively) (Table 3, supplemental Fig. 2, supplemental Table 2 and supplemental Table 3). All secondary outcomes were comparable between the treatment groups: in-hospital mortality (early surgery 26% vs. initial conservative treatment 21%; AOR 0.8 (95% CI, 0.4–1.4)), GOSE 7–8 vs. 1–6 (15% vs. 19%; AOR 1.4 (95% CI, 0.7–2.8)), GOSE 5–8 vs. 1–4 (37% vs. 45%; AOR 0.9 (95% CI, 0.5–1.5)), and GOSE 4–8 vs. 1–3 (42% vs. 51%; AOR 0.8 (95% CI, 0.5–1.3)), “treatment failure” (11% vs. 7%; AOR 1.1 (95% CI, 0.4–3.0)), hospital LOS (median 28 days (IQR 17–54) vs. 21 (IQR 12–42); beta 2.9 (95% CI, − 5.0–10.7)), and Qolibri at 6 months (median 75 (IQR 61–84) vs. 69 (54–80); beta 5.0 (95% CI, − 4.2–14.2)). In the initial conservatively treated group, 14 patients (7%) received cranial surgery after 48 h (i.e., treatment failure). Clinical outcomes of these patients were poor, with a median GOSE at 6 months of 2 (IQR 1,4) (supplemental table 4).

**Table 3:** Primary and secondary outcomes of patients with t-ICH comparing early surgery versus initial conservative treatment

Outcome	Early surgery (*n* = 160)	Initial conservative treatment (*n* = 207)	Effect variable	Unadjusted value (95% CI)	Adjusted value (95% CI)^§^
Primary outcome					
GOSE at 6 months (median (IQR))	2 (1, 4)	3 (1, 5)	Common odds ratio	0.7 (0.5 – 1.0)	1.1 (0.6 – 1.7)
Secondary outcomes					
In-hospital mortality (%)	42 (26)	44 (21)	Odds ratio	1.3 (0.9– 1.9)	0.8 (0.4 – 1.4)
GOSE of 7 or 8 at 6 months (%)	24 (15)	40 (19)	Odds ratio	0.7 (0.5 – 1.2)	1.4 (0.7 – 2.8)
GOSE of 5–8 at 6 months (%)	59 (37)	93 (45)	Odds ratio	0.7 (0.5 – 1.0)	0.9 (0.5 – 1.5)
GOSE of 4–8 at 6 months (%)	67 (42)	106 (51)	Odds ratio	0.7 (0.5 – 1.0)	0.8 (0.5 – 1.3)
Treatment failure*	18 (11)	14 (7)	Odds ratio	1.8 (0.9 – 3.6)	1.1 (0.4 – 3.0)
Qolibri at 6 months (median (IQR))	75 (61, 84)	69 (54, 80)	Beta	4.6 (− 3.4 – 12.6)	5.0 (− 4.2 – 14.2)
Hospital length of stay, days (median (IQR))	28 (17, 54)	21 (12, 42)	Beta	14.3 (6.8 – 21.8)	2.9 (− 5.0 – 10.7)

^*^Treatment failure is defined as patients in the initial conservative treatment group who are operated at a delayed moment or patients in the early surgery group who are operated again. ^§^Adjustment in multivariate regression was performed using the following confounders: age, GCS, pupillary reactivity, midline shift, hematoma size, hematoma unilaterality, and concomitant EDH and ASDH. Abbreviations: *GOSE*, Glasgow Outcome Scale Extended; *IQR*, interquartile range; *CI*, confidence interval; *Qolibri*, Quality of Life after Brain Injury Questionnaire

The sensitivity analyses including patients with infaust prognosis did not show different results (supplemental Table 3). The sensitivity analysis using timing from admission to surgery showed no association of earlier surgery with higher GOSE (supplemental Table 3). Moreover, no difference was found on functional outcome comparing DC versus craniotomy in the early surgery group (supplemental Table 3).

**Fig. 2:**
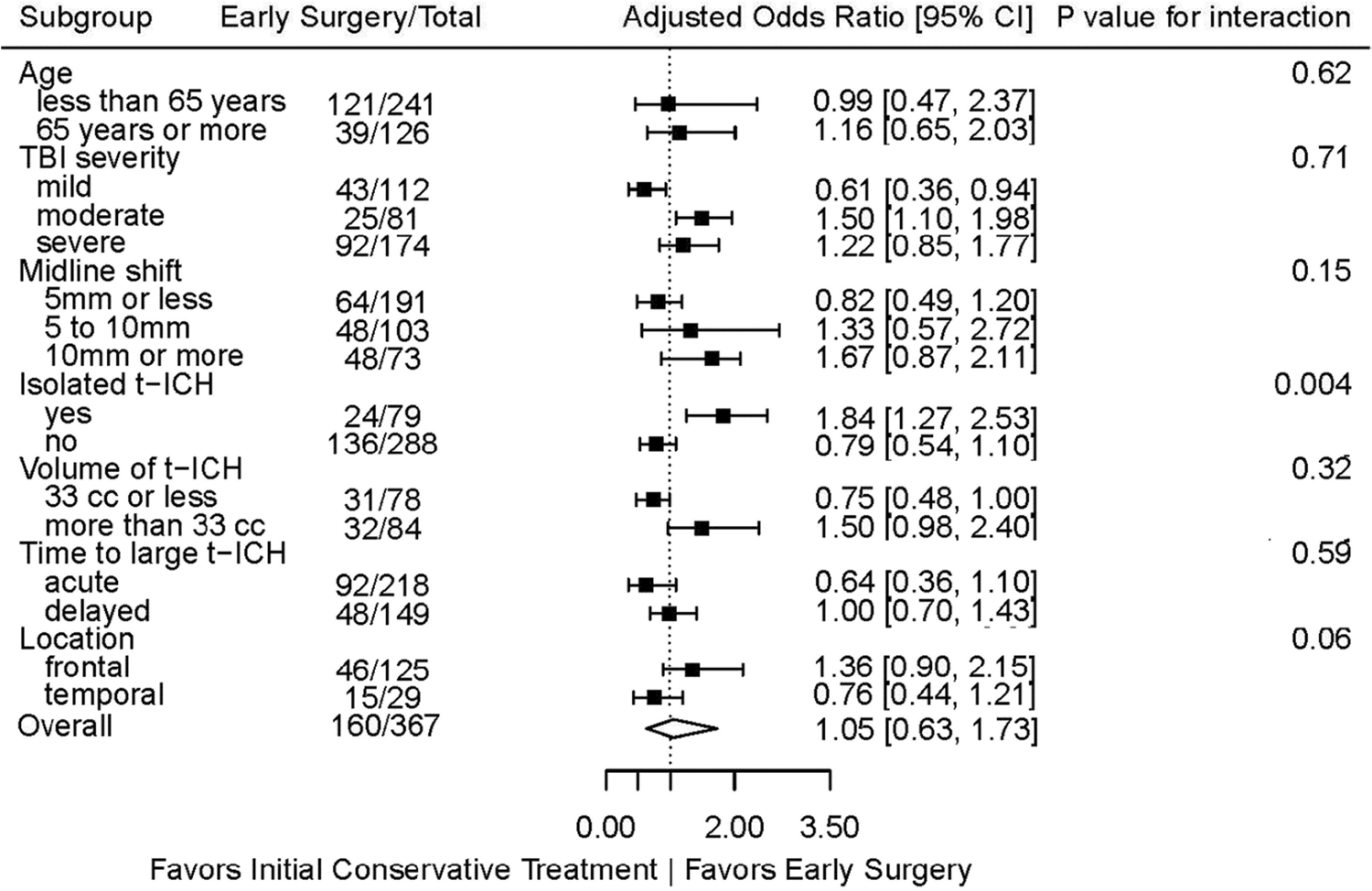
Subgroup analyses of the primary outcome comparing early surgery versus initial conservative treatment. *TBI severity: mild TBI (GCS 15–13), moderate TBI (GCS 9–12), and severe TBI (GCS < 9). **Isolated t-ICH: without concomitant ASDH or EDH. ***Volume of t-ICH: median split of 33 cc used. ****Acute: large t-ICH on admission, decision made after first CT scan. Delayed: blossoming large t-ICH within 48 h of admission (not present on admission), decision made after that specific CT scan. ****Only subgroup analyses performed on location of the largest t-ICH “frontal” and “temporal” as sample size did not allow for analyses on other locations. Abbreviations: CI: confidence interval; TBI: traumatic brain injury; t-ICH: traumatic intracerebral

In subgroup analyses, conservative treatment was associated with better outcome in patients with mild TBI (AOR 0.6 (95% CI, 0.4–0.9); *P* value for interaction 0.71) and a smaller t-ICH (≤ 33 cc) (AOR 0.8 (95% CI, 0.5–1.0); *P* value for interaction 0.32) (Fig. 2). On the other hand, early surgery was associated with better outcome in patients with moderate TBI (GCS 9–12) (AOR 1.5 (95% CI, 1.1–2.0); *P* value for interaction 0.71) and isolated t-ICH (AOR 1.8 (95% CI, 1.3–2.5); *P* value for interaction 0.004). Moreover, early surgery was associated with better outcome in patients with a larger t-ICH (> 33 cc), although not achieving statistical significance (AOR 1.5 (95% CI, 1.0–2.4); *P* value for interaction 0.32).

When comparing patients with isolated t-ICH, without a concomitant EDH or SDH, 24 patients received early surgery, whereas 55 patients were treated conservatively (supplemental table 5). The patients with an isolated t-ICH receiving early surgery were younger (median 31 vs. 64 years) and healthier (ASAPS “healthy” 79% vs. 46%). Radiologic variables, including midline shift and compression of basal cisterns, were not significant different between the two groups. The median volume of the t-ICH was 27 cc in the early surgery group compared to 13 cc in the conservatively treated group.

## Discussion

Early surgery for t-ICH was not associated with improved functional outcome compared to conservative treatment in the overall sample. However, in patients with moderate TBI or an isolated t-ICH, early surgery was associated with improved functional outcome. These results complement results from the STITCH(Trauma) trial [16]. Furthermore, early surgery as compared to conservative treatment was associated with better outcome in patients with a large t-ICH (> 33 cc), while early surgery versus conservative treatment was associated with similar outcome in mild TBI and patients with smaller t-ICH (≤ 33).

RCTs are the gold standard for assessing efficacy of interventions. However, surgical trials in the acute setting can be challenging. They are typically conducted with highly selected patient population, and certain patients are more likely to be randomized than others—as surgical equipoise is not always achieved—resulting in poor generalizability. Therefore, sophisticated observational studies may complement RCTs in order to inform practice [4]. Observational studies, like the current study, closely resemble daily clinical practice by including more heterogeneous patient populations and less stringent treatment and protocols, therefore increasing generalizability, possibly at the expense of the internal validity [7]. Thus, like any observational study, our study leaves the possibility of residual (unmeasured) confounding open, despite the methodology using both regression-based covariate adjustment and PSM [22]. Part of the poor outcome in the early surgery group is likely explained by a relatively larger proportion of patients that would die without surgery. This skewed overrepresentation of an extremely poor prognosis in the surgery group can be deduced by comparison with the mortality risks of the treatment groups of STITCH(trauma): an excess mortality is seen in our surgery group (26 vs. 15%), while relatively fewer patients have died in the conservative treatment group (21 vs. 33%). This also resembles clinical practice where surgery is more often seen as the plausible last resort than conservative treatment. It alludes to the human instinct to act or do something for patients with a life-threatening condition.

Our pre-defined protocol specified IV analysis, thereby “allocating” patients to be exposed to differing likelihoods of receiving surgery. However, our study did not meet the criteria to perform IV analysis. There was insufficient between-center variation to justify the proposed method, and the sample size was not large enough to reliably determine a clinically relevant treatment effect. The relatively limited treatment variation is in line with results of our provider profiling surveys exploring differences in neurosurgical strategies for TBI [21].

Although surgical decision-making might be straightforward in patients with extreme or minimal pathology and clinical signs, the challenge lies in the prognostic “middle” group, for which the patients’ symptoms and pathology are at neither extreme [1, 2, 18]. The inclusion of all t-ICH patients with a large hematoma as judged by the neurosurgeon in our study results in a heterogeneous study sample. This could have led to a neutral treatment effect through averaging out of subgroups effects, a core characteristic of multiple “failing” studies in TBI research [13, 14]. Thus, although no overall beneficial association of surgery was found in our study, this should not be interpreted as the absence of a treatment effect. Subgroup analyses indicate that treatment effects differ within various TBI subgroups, and some patients seem to benefit from either one of the treatment strategies.

To provide a complementary body of evidence that optimizes both internal and external validity on which to base surgical decision-making, findings from our study should be compared to those from the STITCH(Trauma) trial. Treatment effect estimates can be influenced by differences between the two patient populations. Our study population was older, with lower GCS, more pupil abnormalities, and a larger t-ICH volume at baseline. Most importantly, we included patients with a concomitant EDH and ASDH in primary analysis, whereas those patients were excluded in the STITCH(Trauma) study. Our subgroup analysis of patients with isolated large t-ICH, excluding those with a concomitant EDH and/or ASDH, resulted in a more favorable effect of early surgery. This subgroup might best represent the STITCH(Trauma) study population and indicates that the beneficial effect of early surgery in patients with a large and isolated t-ICH holds true in daily clinical practice. Moreover, our results confirm the benefit of early surgery in patients with GCS 9–12. Our study found similar results to the STITCH(Trauma) trial using various statistical methods, indicating that there could be a role for early surgery in patients with an isolated t-ICH and those with a baseline GCS 9–12.

To our knowledge, the current study is the first to explore the representativeness of the benefit of early surgery in t-ICH found in the STITCH(Trauma) trial, using the hitherto largest sample. Nevertheless, we have to acknowledge a few limitations. First, on primary analysis, we included patients with a concomitant EDH/ASDH, which might result in confusion of surgical indications when the primary reason for surgery was an EDH/ASDH. However, a subgroup analysis of patients with isolated t-ICH was performed. Second, potential residual confounding and selection bias inherent to the observational design cannot be ruled out. Although sensitivity analyses did not alter the overall effect estimate confirming the robustness of our primary analysis, there is still the possibility of confounding within subgroups, leading to type I error in these analyses. Finally, the relatively small samples for the subgroup analyses and the selective participation of neurotrauma oriented centers may have impacted the generalizability.

## Conclusion

Patients with large t-ICH, including those with isolated t-ICH and moderate TBI, might benefit from early surgery, compatible with the effect observed in the STITCH(Trauma) trial.

### Supplementary Information

Below is the link to the electronic supplementary material.

Supplementary file1 (DOCX 680 KB)

## References

[1] Bullock MR (2006). Surgical management of traumatic parenchymal lesions. Neurosurgery.

[2] Bullock R, Golek J, Blake G (1989). Traumatic intracerebral hematoma–which patients should undergo surgical evacuation? CT scan features and ICP monitoring as a basis for decision making. Surg Neurol.

[3] Gregson BA, Mitchell P, Mendelow AD (2019). Surgical decision making in brain hemorrhage. Stroke.

[4] He Z (2020). Clinical trial generalizability assessment in the big data era: a review. Clin Transl Sci.

[5] Hernán MA, Robins JM (2016). Using big data to emulate a target trial when a randomized trial is not available. Am J Epidemiol.

[6] Houvenaeghel G (2020). External validation of the SERC trial population: comparison with the Multicenter French Cohort, the Swedish and SENOMIC trial populations for breast cancer patients with sentinel node micro-metastasis. Cancers (Basel).

[7] Kennedy-Martin T (2015). A literature review on the representativeness of randomized controlled trial samples and implications for the external validity of trial results. Trials.

[8] Lesko CR (2020). Target validity: bringing treatment of external validity in line with internal validity. Curr Epidemiol Rep.

[9] Lingsma HF (2010). Statin treatment after a recent TIA or stroke: is effectiveness shown in randomized clinical trials also observed in everyday clinical practice?. Acta Neurol Scand.

[10] Lingsma H (2013). Prognosis in moderate and severe traumatic brain injury: external validation of the IMPACT models and the role of extracranial injuries. J Trauma Acute Care Surg.

[11] Maas AI (2015). Collaborative European neurotrauma effectiveness research in traumatic brain injury (CENTER-TBI): a prospective longitudinal observational study. Neurosurgery.

[12] Maas AIR (2017). Traumatic brain injury: integrated approaches to improve prevention, clinical care, and research. Lancet Neurol.

[13] Maas A, Stocchetti N (2011). Hypothermia and the complexity of trials in patients with traumatic brain injury. Lancet Neurol.

[14] Maas AI, Roozenbeek B, Manley GT (2010). Clinical trials in traumatic brain injury: past experience and current developments. Neurotherapeutics.

[15] Maciejewski ML, Brookhart MA (2019). Using instrumental variables to address bias from unobserved confounders. JAMA.

[16] Mendelow AD (2015). Early surgery versus initial conservative treatment in patients with traumatic intracerebral hemorrhage (STITCH[Trauma]): the first randomized trial. J Neurotrauma.

[17] Nelson KS, Brearley AM, Haines SJ (2014). Evidence-based assessment of well-established interventions: the parachute and the epidural hematoma. Neurosurgery.

[18] Posti JP, Raj R, Luoto TM (2020) How do we identify the crashing traumatic brain injury patient - the neurosurgeon's view. Curr Opin Crit Care 10.1097/MCC.0000000000000799 33395087

[19] Rothwell PM (2005). External validity of randomised controlled trials: "to whom do the results of this trial apply?". Lancet.

[20] Van Essen TA (2019). Comparative effectiveness of surgery in traumatic acute subdural and intracerebral haematoma: study protocol for a prospective observational study within CENTER-TBI and Net-QuRe. BMJ Open.

[21] van Essen TA (2019). Variation in neurosurgical management of traumatic brain injury: a survey in 68 centers participating in the CENTER-TBI study. Acta Neurochir (Wien).

[22] van Essen TA, Menon DK, Lingsma HF (2020). Unmeasured confounding in observational studies of management of cerebellar intracranial hemorrhage. JAMA.

[23] von Elm E (2007). The Strengthening the Reporting of Observational Studies in Epidemiology (STROBE) statement: guidelines for reporting observational studies. Lancet.

[24] von Steinbuechel N, Petersen C, Bullinger M (2005). Assessment of health-related quality of life in persons after traumatic brain injury–development of the Qolibri, a specific measure. Acta Neurochir Suppl.

[25] Wilson JT, Pettigrew LE, Teasdale GM (1998). Structured interviews for the Glasgow Outcome Scale and the extended Glasgow Outcome Scale: guidelines for their use. J Neurotrauma.

[26] Wu E, Marthi S, Asaad WF (2020). Predictors of mortality in traumatic intracranial hemorrhage: a National Trauma Data Bank study. Front Neurol.

