# Supplementary material for: Early surgery versus conservative treatment in patients with traumatic intracerebral hematoma: a CENTER-TBI study
Source: Acta Neurochir (Wien). 2023 Sep 25;165(11):3217–27. doi: 10.1007/s00701-023-05797-y (PMC10624744; doi:10.1007/s00701-023-05797-y)

**Supplement**

Table 1. Baseline and radiological characteristics of patients with a moribund prognosis

Figure 1a. Physicians’ motivations for early surgery and initial conservative treatment on study cohort

Figure 1b. Physicians’ motivations for early surgery and initial conservative treatment on study cohort

Figure 2. Kernel Density plot of propensity scores

Table 2. Baseline and radiological characteristics of propensity matched cohort, comparing early surgery versus initial conservative treatment

Table 3. Results of sensitivity analyses: covariable adjustment, propensity score matching, timing to surgery and decompressive craniectomy vs. craniotomy with the Glasgow Outcome Scale Extended as outcome

Figure 3. Volume of traumatic intracerebral hematomas per center

Figure 4. Between-center differences in early surgery

Table 4. Outcome characteristics of patients with delayed surgery (>48 hours) in the initial conservative treatment group

Table 5. Baseline and radiological characteristics of patients with isolated t-ICH (no concomitant hematomas) comparing early surgery versus initial conservative treatment

Figure 5a. Physicians’ motivations for early surgery on predefined subgroups

Figure 5b. Physicians’ motivations for initial conservative treatment on

predefined subgroups

**Table 1. Baseline and radiological characteristics of patients with a moribund prognosis**

|  | **Moribund prognosis patients** | **Missing (%)** |
| --- | --- | --- |
| n | 59 |  |
| Age (median [IQR]) | 70 [58, 78] | 0 |
| Male (%) | 37 (63) | 0 |
| Cause of injury (%) |  | 3 |
| Road traffic incident | 15 (25) |  |
| Incidental fall | 36 (61) |  |
| Other non-intentional injury | 1 (2) |  |
| Assault/violence | 1 (2) |  |
| Suicide attempt | 3 (5) |  |
| Other | 1 (2) |  |
| ASAPS (%) |  | 12 |
| Healthy | 8 (14) |  |
| Mild systemic disease | 28 (48) |  |
| Severe systemic disease | 15 (25) |  |
| Threat to life | 1 (2) |  |
| Antithrombotic medication (%) |  | 12 |
| No | 26 (44) |  |
| Yes, anticoagulants | 7 (12) |  |
| Yes, platelet aggregation inhibitors | 19 (32) |  |
| Hypoxia (%)^§^ |  | 7 |
| No | 48 (81) |  |
| Definite | 6 (10) |  |
| Suspect | 1 (2) |  |
| Hypotension (%)^§^ |  | 7 |
| No | 49 (83) |  |
| Definite | 5 (6) |  |
| Suspect | 1 (2) |  |
| GCS (median [IQR]) | 5 [3, 10] | 2 |
| GCS motor (median [IQR]) | 1 [1, 5] | 0 |
| Pupils (%) |  | 3 |
| Both reacting | 25 (44) |  |
| One reacting | 4 (7) |  |
| Both unreacting | 28 (49) |  |
| ISS (median [IQR]) | 43 [25, 75] | 0 |
| AIS head (median [IQR]) | 5 [5, 6] | 0 |
| TBI severity (%)^∞^ |  | 2 |
| Mild | 7 (12) |  |
| Moderate | 9 (16) |  |
| Severe | 42 (72) |  |
| Epidural hematoma (%)^α^ |  | 0 |
| No | 52 (88) |  |
| Small | 5 (9) |  |
| Large | 2 (3) |  |
| Acute subdural hematoma (%)^α^ |  | 0 |
| No | 10 (17) |  |
| Small | 20 (34) |  |
| Large | 29 (49) |  |
| Subarachnoid hemorrhage (%) |  | 0 |
| No | 12 (20) |  |
| Basal | 6 (10) |  |
| Cortical | 20 (34) |  |
| Basal and Cortical | 21 (36) |  |
| Depressed skull fracture (%) |  | 0 |
| No | 44 (75) |  |
| Closed | 12 (20) |  |
| Open | 3 (5) |  |
| Diffuse axonal injury (%) |  | 10 |
| No | 46 (78) |  |
| Yes | 7 (12) |  |
| Midline shift (%)^λ^ | 40 (68) | 0 |
| Midline shift, mm (median [IQR]) | 10 [6, 16] | 37 |
| Compressed basal cisterns (%) | 34 (58) | 0 |
| Volume of largest t-ICH (cc) (median [IQR]) | 34 [20, 103] | 59 |
| Location of largest t-ICH (%) |  | 59 |
| frontal | 19 (79) |  |
| temporal | 4 (17) |  |
| other | 1 (4) |  |
| Two or more t-ICHs (%) | 18 (75) | 59 |
| All t-ICHs unilateral (%) | 7 (29) | 59 |
| Two or more regions involved (%) | 19 (79) | 59 |
| Predicted probability of 6 month mortality (median [IQR])^*^ | 69 [56, 81] | 17 |
| Predicted probability of 6 month unfavourable outcome (median [IQR])^*^ | 82 [74, 92] | 17 |

^§^Definite hypoxia is defined as a documented PaO2 < 8 kPA (60 mmg Hg) and/or SaO2 <90% in pre-hospital or ER phase. Suspected hypoxia was scored if the patient did not have documented hypoxia by PaO2 or SaO2, but there was a clinical suspicion, as evidenced by for example cyanosis, apnoea or respiratory distress

^¥^Definite hypotension is defined as a documented systolic BP < 90 mm Hg in pre-hospital or ER phase. Suspected hypotension was scored if the patient did not have a documented low BP, but was reported to be in shock or have an absent brachial pulse (not related to injury of the extremity)

^∞^Classified as mild TBI (GCS 15-13), moderate TBI (GCS 9-12) and severe TBI (GCS < 9)

^α^Small and large as judged by the treating physician

^λ^Presence of midline shift is classified as being more than 5 mm.

^*^ Calculated using the International Mission for Prognosis and Analysis of Clinical Trials in TBI (IMPACT) score (core model). Percentage missing include those with a GCS > 12

*Abbreviations*: AIS, Abbreviated Injury Scale; ASAPS, American Society of Anesthesiologists classification system; GCS, Glasgow Coma Scale; IQR, interquartile range; ISS: Injury Severity Score; SMD: Standardized mean difference; t-ICH: traumatic intracerebral hematoma

**Figure 1a. Physicians’ motivations for early surgery and initial conservative treatment on study cohort**

**
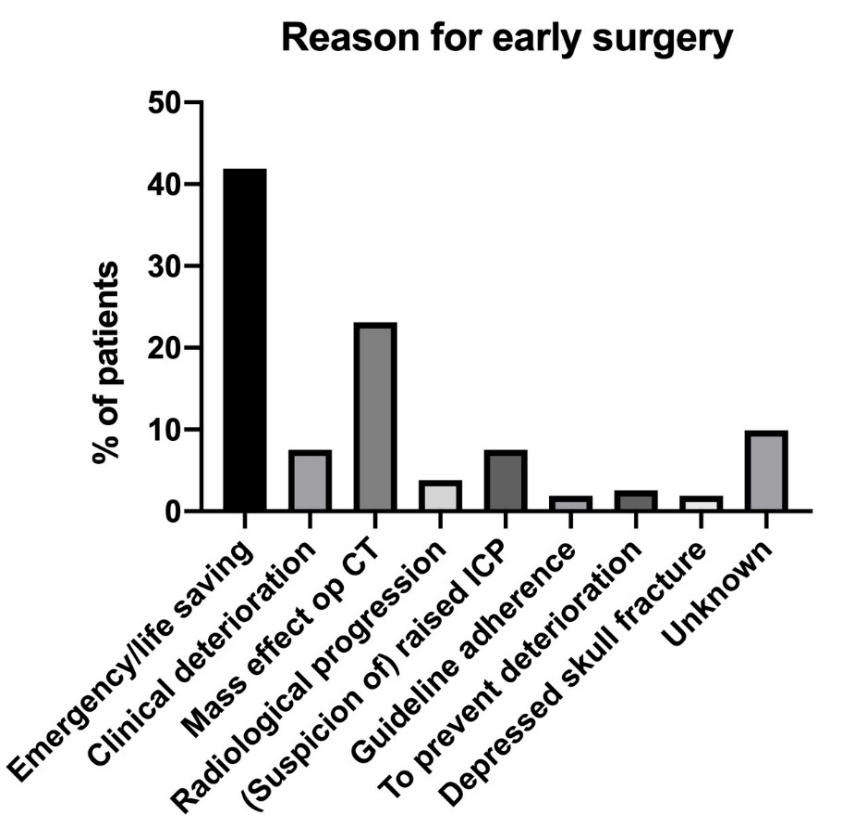
**

**Figure 1b. Physicians’ motivations for early surgery and initial conservative treatment on study cohort**

**
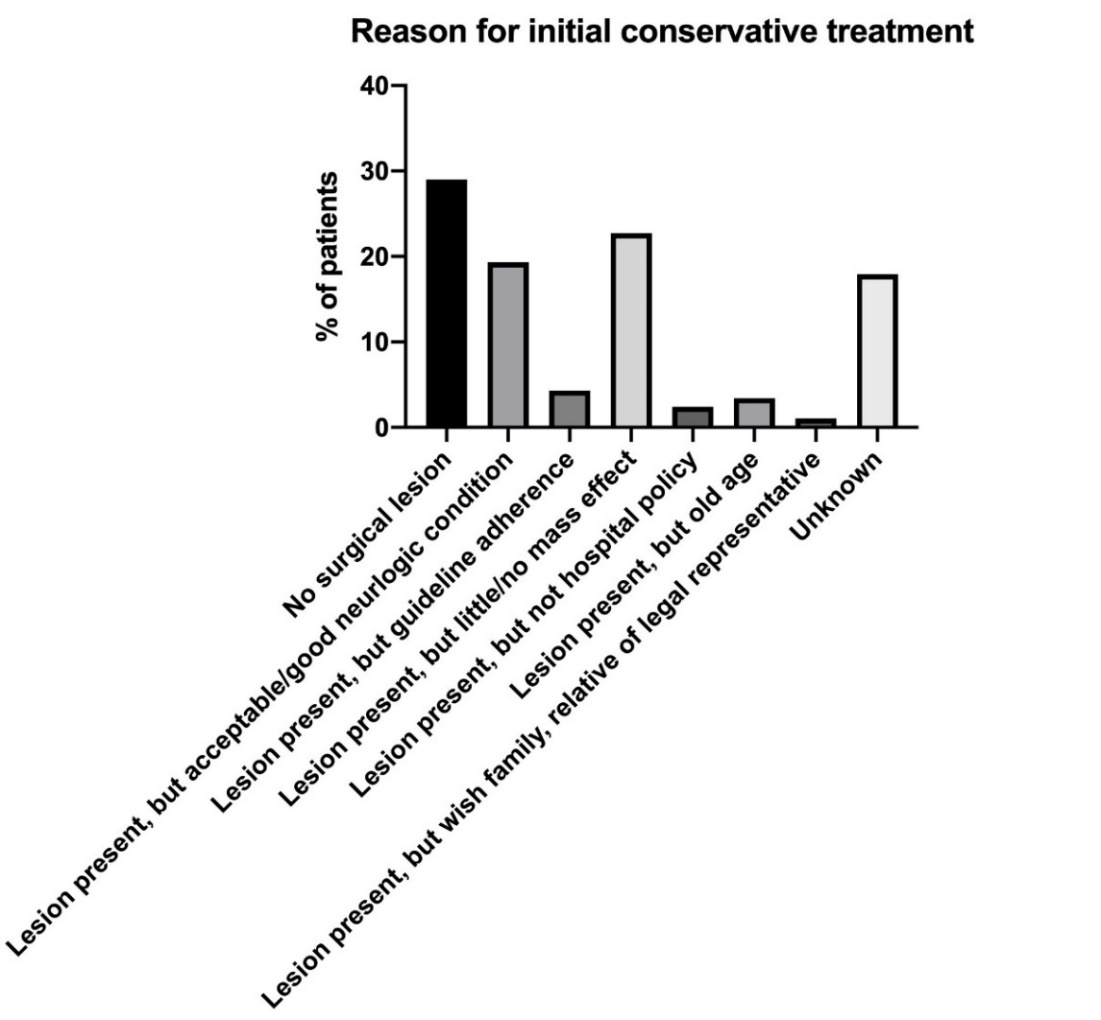
**

**Figure 2. Kernel Density plot of propensity scores**

**
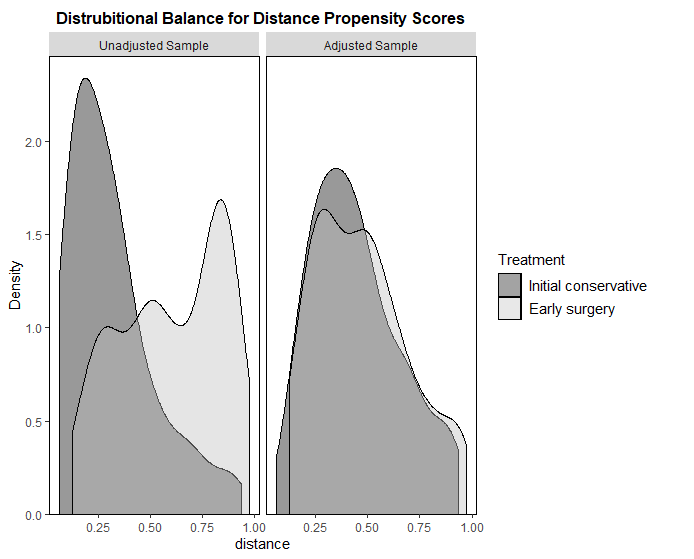
**

**Table 2. Baseline and radiological characteristics of propensity matched cohort, comparing early surgery versus initial conservative treatment**

|  | **Early Surgery** | **Initial Conservative Treatment** | **SMD** |
| --- | --- | --- | --- |
| n | 98 | 98 |  |
| Age (median [IQR]) | 55 [35, 64] | 48 [36, 66] | 0.0 |
| Male (%) | 77 (79) | 73 (75) | 0.1 |
| Predicted probability of 6 month mortality (median [IQR])^*^ | 33 [21, 62] | 27 [20, 51] | 0.2 |
| Predicted probability of 6 month unfavourable outcome (median [IQR])^*^ | 54 [35, 76] | 46 [34, 71] | 0.1 |
| Cause of injury (%) |  |  | 0.3 |
| Road traffic incident | 27 (28) | 31 (32) |  |
| Incidental fall | 48 (49) | 49 (50) |  |
| Other non-intentional injury | 4 (4) | 1 (1) |  |
| Assault/violence | 5 (5) | 7 (7) |  |
| Suicide attempt | 3 (3) | 2 (2) |  |
| Other | 2 (2) | 3 (3) |  |
| ASAPS (%) |  |  | 0.4 |
| Healthy | 60 (61) | 48 (49) |  |
| Mild systemic disease | 22 (22) | 37 (38) |  |
| Severe systemic disease | 10 (10) | 10 (10) |  |
| Antithrombotic medication (%) |  |  | 0.1 |
| No | 82 (84) | 82 (84) |  |
| Yes, anticoagulants | 4 (4) | 5 (5) |  |
| Yes, platelet aggregation inhibitors | 5 (5) | 5 (5) |  |
| Yes, both | 1 (1) | 2 (2) |  |
| GCS (median [IQR]) | 8 [3, 13] | 8 [5, 12] | 0.1 |
| GCS motor (median [IQR]) | 4 [1, 6] | 4 [1, 5] | 0.2 |
| Pupil reactivity (%) |  |  | 0.1 |
| Both reacting | 75 (77) | 73 (75) |  |
| One reacting | 10 (10) | 8 (8) |  |
| Both unreacting | 13 (13) | 17 (17) |  |
| Any major extracranial injury^¥^ | 43 (44) | 51 (52) | 0.2 |
| Epidural hematoma (%)^α^ |  |  | 0.2 |
| No | 78 (80) | 82 (84) |  |
| Small | 11 (11) | 11 (11) |  |
| Large | 9 (9) | 5 (5) |  |
| Acute subdural hematoma (%)^α^ |  |  | 0.1 |
| No | 29 (30) | 31 (32) |  |
| Small | 42 (43) | 45 (46) |  |
| Large | 27 (28) | 22 (22) |  |
| Subarachnoid hemorrhage (%) |  |  | 0.3 |
| No | 16 (16) | 26 (27) |  |
| Basal | 11 (11) | 11 (11) |  |
| Cortical | 54 (55) | 43 (44) |  |
| Basal and Cortical | 17 (17) | 18 (18) |  |
| Midline shift, mm (median [IQR]) | 7 [4, 12] | 5.0 [3.0, 7.0] | 0.3 |
| Volume of largest t-ICH (cc) (median [IQR]) | 44 [16, 78] | 41.6 [13, 88] | 0.0 |
| Location of largest t-ICH (%) |  |  | 0.5 |
| frontal | 47 (48) | 33 (34) |  |
| temporal | 34 (35) | 28 (29) |  |
| occipital | 7 (7) | 8 (8) |  |
| parietal | 6 (6) | 21 (21) |  |
| All t-ICHs unilateral (%) | 52 (53) | 53 (54) | 0.0 |

^*^ Calculated using the International Mission for Prognosis and Analysis of Clinical Trials in TBI (IMPACT) score (core model). Percentage missing include those with a GCS > 12

^¥^Any major extracranial injury is classified as one or more body region(s) with AIS > 3 (except for AIS head)

^α^Small and large as judged by the treating physician

^λ^Presence of midline shift is classified as being more than 5 mm.

*Abbreviations*: AIS, Abbreviated Injury Scale; ASAPS, American Society of Anesthesiologists classification system; GCS, Glasgow Coma Scale; IQR, interquartile range; SMD: Standardized mean difference; t-ICH: traumatic intracerebral hematoma

**Table 3. Results of sensitivity analyses: covariable adjustment, propensity score matching, timing to surgery and decompressive craniectomy vs. craniotomy with the Glasgow Outcome Scale Extended as outcome**

| ***Approach*** | ***Early surgery (OR 95 % CI)*** |
| --- | --- |
| Unadjusted model (study cohort) | 0.7 (0.5 – 1.0) |
| Covariable adjustment ^a^ |  |
| Study cohort | 1.1 (0.6 – 1.7) |
| Complete cohort (moribund prognosis = ICT and GOSE = 1) ^b^ | 1.5 (0.9 – 2.7) |
| Complete cohort (moribund prognosis = ES and GOSE = 1) ^b^ | 0.7 (0.5 – 1.2) |
| Complete cohort (moribund prognosis = ICT and GOSE = 8) ^b^ | 0.8 (0.5 – 1.2) |
| Complete cohort (moribund prognosis = ES and GOSE = 8) ^b^ | 1.5 (0.8 – 2.4) |
| Propensity score matching ^c^ | 1.1 (0.8 – 1.5) |
|  |  |
|  | ***Timing to surgery ^d^ (OR 95 % CI)*** |
| Unadjusted model | 1.0 (0.5 – 1.9) |
| Covariable adjustment ^a^ | 1.0 (0.6 – 1.7) |
|  |  |
|  | ***Decompressive craniectomy vs craniotomy^e^  (OR 95 % CI)*** |
| Unadjusted model | 0.5 (0.3 – 0.7) |
| Covariable adjustment ^a^ | 1.4 (0.7 – 1.9) |

^a^ Model was adjusted for the following confounders: age, GCS, pupillary reactivity, midline shift, hematoma size, and concomitant EDH and ASDH

^b^ Complete cohort analyses includes the patients with a moribund prognosis (eTable 1).

^c^ A propensity score was calculated based on the following variables: age, GCS, pupillary reactivity, midline shift, hematoma size, and concomitant EDH and ASDH

^d^ Timing to surgery is defined as a continuous variables ‘time from admission to inclusion/surgery’, including early surgery patients who had surgery and initial conservative treatment patients who required delayed surgery, therefore independent of treatment group.

^e^ Analyses of the patients in the early surgery group.

*Abbreviations*: CI, confidence interval; ES, early surgery; ICT, initial conservative treatment; OR, odds ratio.

**Figure 3. Volume of traumatic intracerebal hematomas per center**


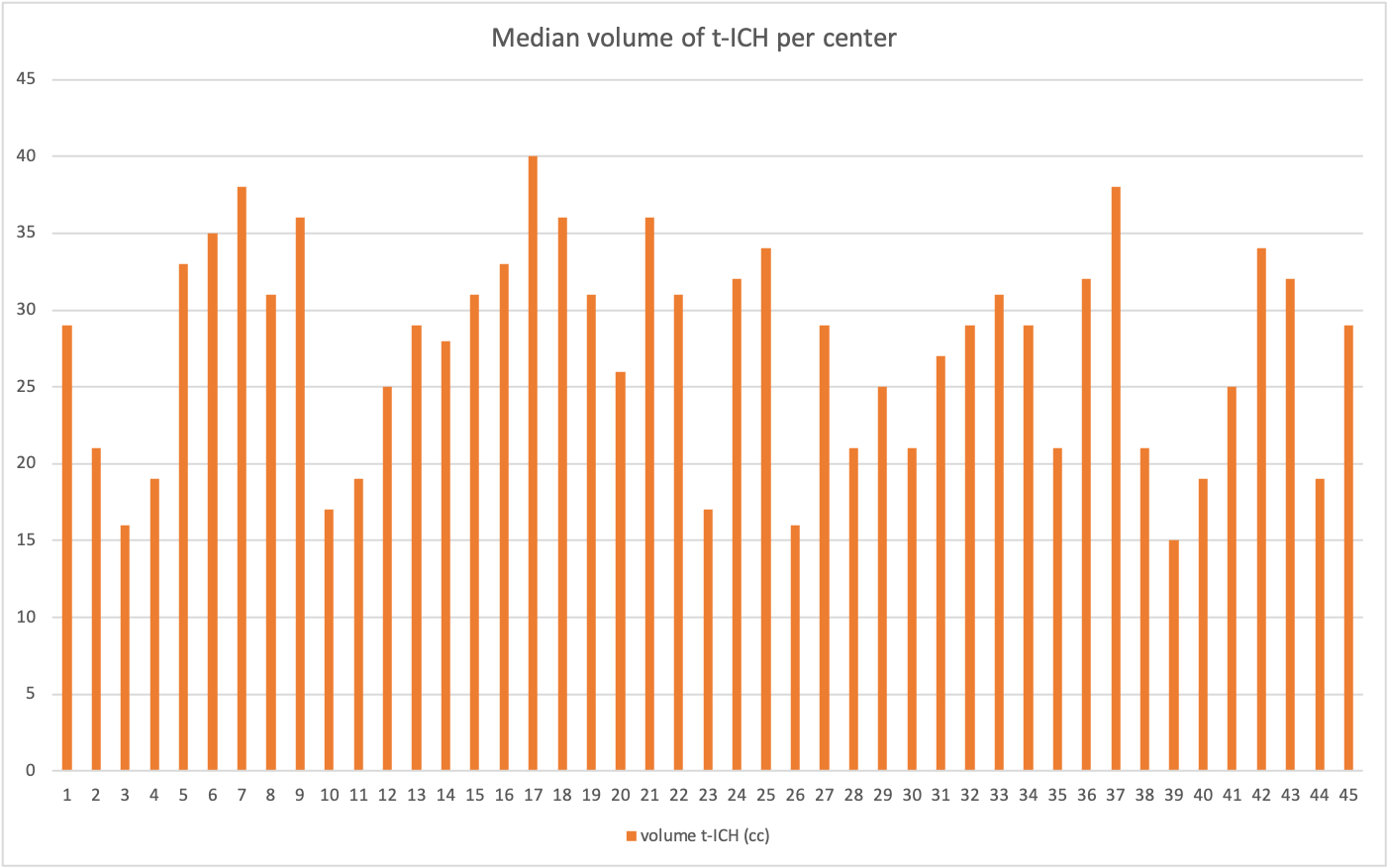


SMD between centres 0.1, ANOVA test p-value 0.34.

**Figure 4. Between-center differences in early surgery**

**
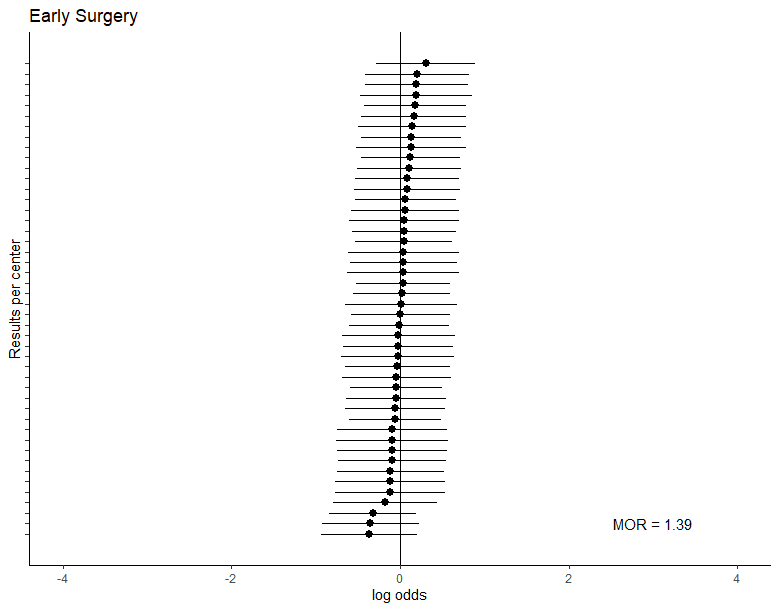
**

The median odds ratio (MOR) reflects the between-center variation; a MOR equal to 1 represents no variation, the larger the MOR, the larger the variation. The MOR is non-significant (p = 0.27)

**Table 4. Outcome characteristics of patients with delayed surgery (>48 hours) in the initial conservative treatment group**

|  | Delayed surgery after initially conservative treatment (n=14) |
| --- | --- |
| Primary outcome |  |
| GOSE at 6 months [median (IQR)] | 2 [1, 4] |
|  |  |
| Secondary outcome |  |
| In-hospital mortality (%) | 2 (14) |
| Qolibri at 6 months [median (IQR)] | 39 [23, 55] |
| Hospital length of stay [median (IQR)] | 46 [21, 64] |

*Abbreviations*: GOSE: Glasgow outcome scale extended; IQR, interquartile range; Qolibri: quality of life after brain injury

**Table 5. Baseline and radiological characteristics of patients with isolated t-ICH (no concomitant hematomas) comparing early surgery versus initial conservative treatment**

|  | **Early surgery** | **Initial conservative treatment** | **SMD** | **Missing** |
| --- | --- | --- | --- | --- |
| **n** | 24 | 55 |  |  |
| **Age (median [IQR])** | 31 [24, 48] | 64 [36, 71] | 0.3 | 0 |
| **Male (%)** | 20 (83) | 44 (67) | 0.2 | 0 |
| **Cause of injury (%)** |  |  | 0.4 | 0 |
| **Road traffic incident** | 11 (46) | 24 (37) |  |  |
| **Incidental fall** | 8 (33) | 29 (45) |  |  |
| **Other non-intentional injury** | 1 (4) | 3 (5) |  |  |
| **Assault/violence** | 2 (8) | 1 (2) |  |  |
| **Other** | 1 (4) | 3 (5) |  |  |
| **ASAPS (%)** |  |  | 0.4 | 0 |
| **Healthy** | 19 (79) | 30 (46) |  |  |
| **Mild systemic disease** | 2 (8) | 26 (40) |  |  |
| **Severe systemic disease** | 3 (13) | 7 (11) |  |  |
| **Antithrombotic medication (%)** |  |  | 0.4 | 0 |
| **No** | 21 (88) | 42 (65) |  |  |
| **Yes, anticoagulants** | 0 (0) | 6 (9) |  |  |
| **Yes platelet aggregation inhibitors** | 3 (13) | 14 (22) |  |  |
| **Hypoxia (%)**^§^ |  |  | 0.6 | 0 |
| **No** | 20 (83) | 20 (83) |  |  |
| **Definite** | 4 (17) | 3 (5) |  |  |
| **Suspect** | 0 (0) | 3 (5) |  |  |
| **Hypotension (%)**^¥^ |  |  | 0.5 | 0 |
| **No** | 15 (63) | 55 (85) |  |  |
| **Definite** | 6 (25) | 5 (8) |  |  |
| **Suspect** | 1 (4) | 1 (2) |  |  |
| **GCS (median [IQR])** | 7 [4, 11] | 9 [4, 13] | 0.2 | 1 |
| **GCS motor (median [IQR])** | 3 [1, 5] | 5 [1, 6] | 0.5 | 1 |
| **Pupil reactivity (%)** |  |  | 0.5 | 7 |
| **Both reacting** | 17 (74) | 50 (83) |  |  |
| **One reacting** | 3 (13) | 3 (5) |  |  |
| **Both unreacting** | 3 (13) | 7 (12) |  |  |
| **ISS (median [IQR])** | 32 [25, 50] | 26 [22, 42] | 0.3 | 0 |
| **AIS head (median [IQR])** | 5 [5, 5] | 5 [4, 5] | 0.8 | 0 |
| **TBI severity (%)**^∞^ |  |  | 0.3 | 1 |
| **Mild** | 5 (22) | 20 (31) |  |  |
| **Moderate** | 5 (22) | 14 (22) |  |  |
| **Severe** | 13 (57) | 31 (48) |  |  |
| **Subarachnoid hemorrhage (%)** |  |  | 0.7 | 0 |
| **No** | 5 (21) | 29 (45) |  |  |
| **Basal** | 2 (8) | 3 (5) |  |  |
| **Cortical** | 15 (63) | 22 (34) |  |  |
| **Basal and Cortical** | 2 (8) | 11 (17) |  |  |
| **Depressed skull fracture (%)** |  |  | 0.7 | 0 |
| **No** | 14 (58) | 52 (80) |  |  |
| **Closed** | 5 (21) | 8 (12) |  |  |
| **Open** | 5 (21) | 5 (8) |  |  |
| **Diffuse axonal injury (%)** |  |  | 0.4 | 5 |
| **No** | 18 (75) | 51 (79) |  |  |
| **Yes** | 5 (21) | 9 (14) |  |  |
| **Midline shift (%)**^λ^ | 12 (50) | 18 (28) | 0.7 | 0 |
| **Midline shift, mm (median [IQR])** | 6 [5, 7] | 6 [4, 8] | 0.9 | 69 |
| **Compressed basal cisterns (%)** | 10 (42) | 16 (25) | 0.3 | 1 |
| **Volume of largest t-ICH (cc) (median [IQR])** | 27 [14, 35] | 13 [5, 33] | 0.8 | 6 |
| **Two or more t-ICHs (%)** | 5 (25) | 12 (27) | 0.3 | 6 |
| **All_t-ICHs_unilateral (%)** | 6 (32) | 7 (19) | 0.8 | 6 |
| **Two or more regions involved (%)** | 7 (35) | 15 (38) | 0.1 | 6 |
| **Location of largest t-ICH (%)** |  |  | 0.1 | 6 |
| **frontal** | 7 (35) | 15 (38) |  |  |
| **occipital** | 1 (8) | 0 (0) |  |  |
| **parietal** | 1 (8) | 1 (5) |  |  |
| **temporal** | 0 (0) | 4 (14) |  |  |
| **Predicted probability of 6 month mortality (median [IQR])**^*^ | 45 [11, 63] | 41 [29, 55] | 0.3 | 35 |
| **Predicted probability of 6 month unfavourable outcome (median [IQR])**^*^ | 62 [22, 76] | 59 [47, 70] | 0.3 | 35 |

^§^Definite hypoxia is defined as a documented PaO2 < 8 kPA (60 mmg Hg) and/or SaO2 <90% in pre-hospital or ER phase. Suspected hypoxia was scored if the patient did not have documented hypoxia by PaO2 or SaO2, but there was a clinical suspicion, as evidenced by for example cyanosis, apnoea or respiratory distress

^¥^Definite hypotension is defined as a documented systolic BP < 90 mm Hg in pre-hospital or ER phase. Suspected hypotension was scored if the patient did not have a documented low BP, but was reported to be in shock or have an absent brachial pulse (not related to injury of the extremity)

^∞^Classified as mild TBI (GCS 15-13), moderate TBI (GCS 9-12) and severe TBI (GCS < 9)

^α^Small and large as judged by the treating physician

^λ^Presence of midline shift is classified as being more than 5 mm.

^*^ Calculated using the International Mission for Prognosis and Analysis of Clinical Trials in TBI (IMPACT) score (core model). Percentage missing include those with a GCS > 12

*Abbreviations*: AIS, Abbreviated Injury Scale; ASAPS, American Society of Anesthesiologists classification system; GCS, Glasgow Coma Scale; IQR, interquartile range; ISS: Injury Severity Score; SMD: Standardized mean difference; t-ICH: traumatic intracerebral hematoma


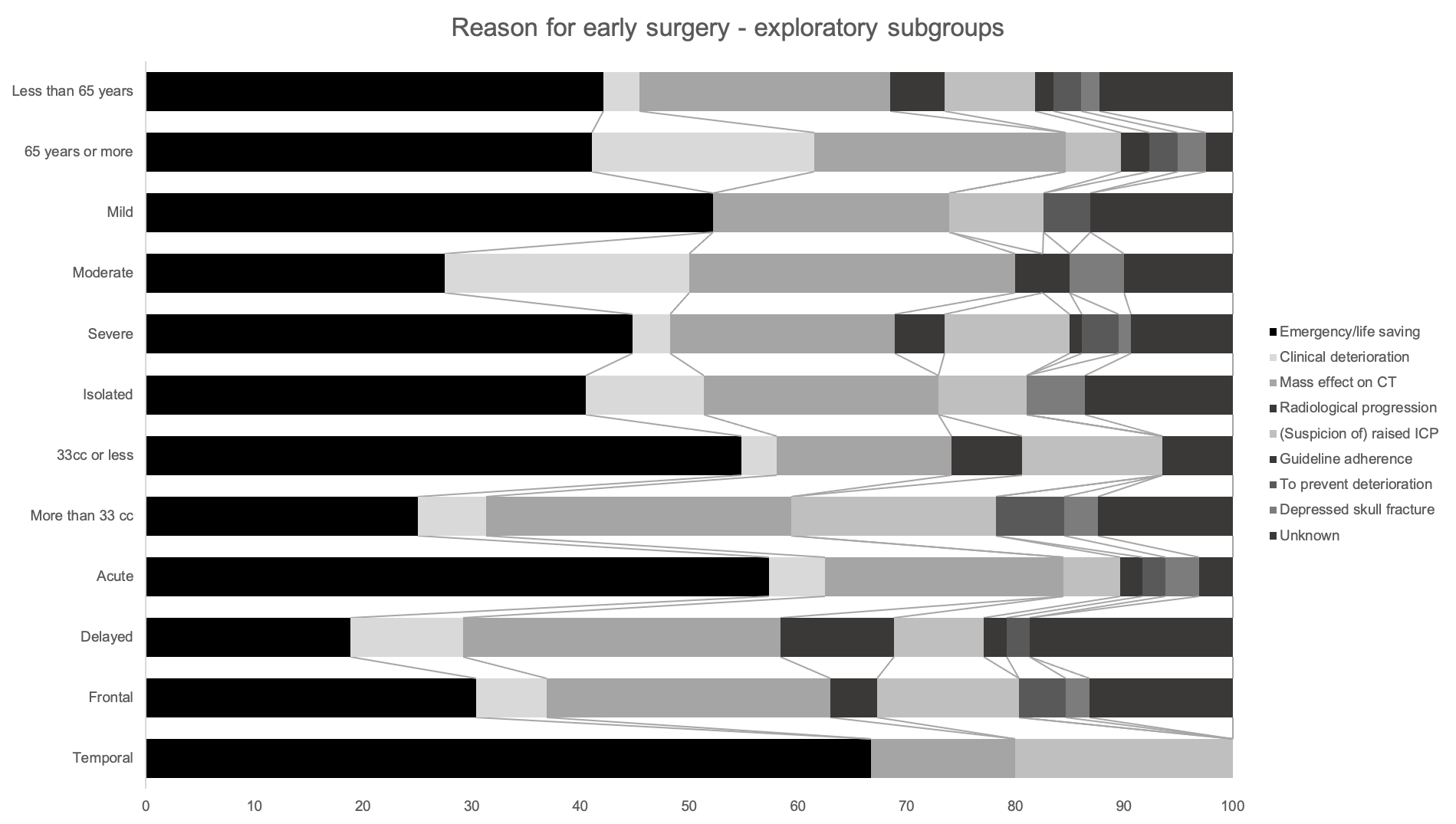
 **Figure 5a. Physicians’ motivations for early surgery on predefined subgroups**

**Figure 5b. Physicians’ motivations for conservative treatment on predefined subgroups**


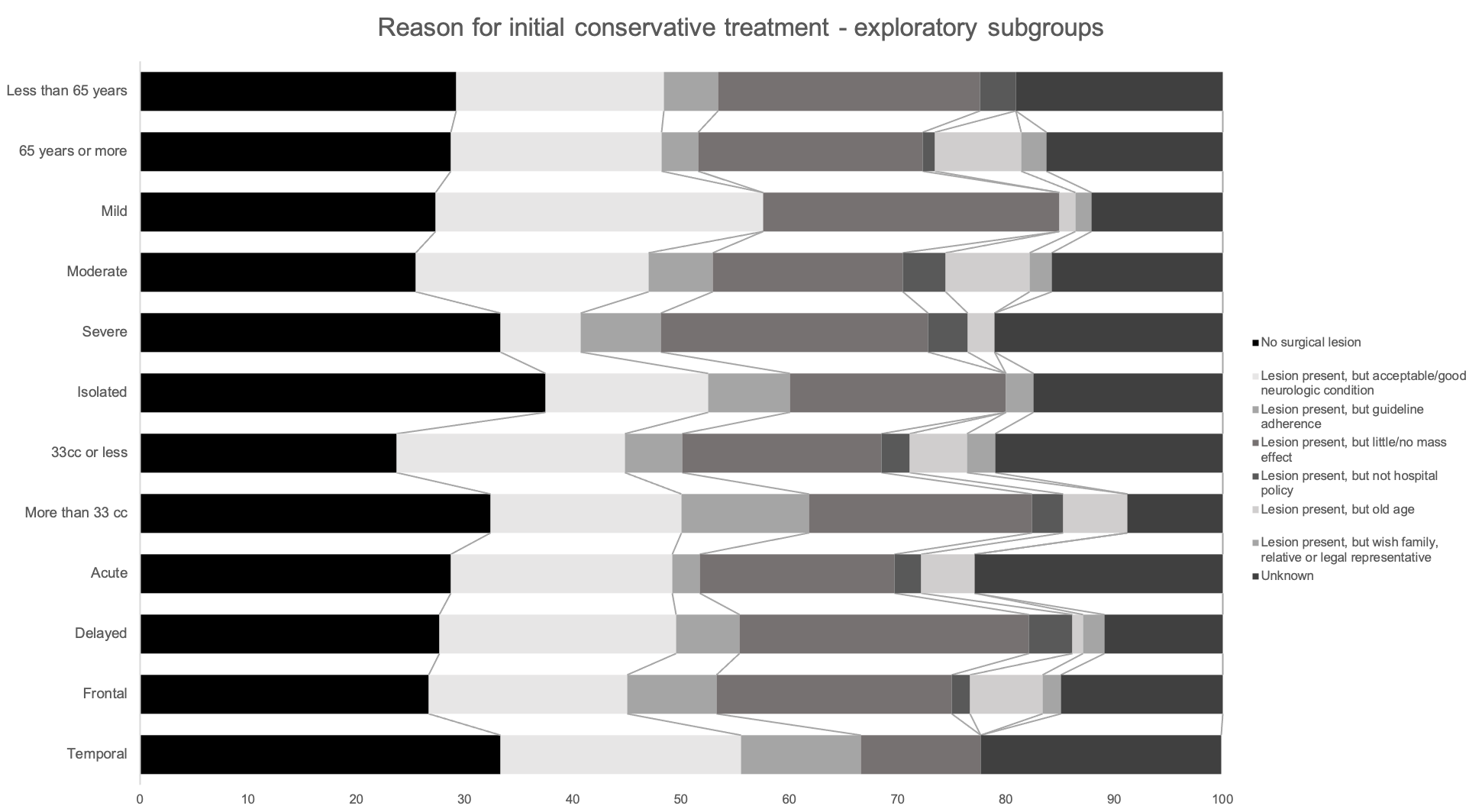

Supplement: Supplementary file 1 — Supplementary file1 (DOCX 680 KB) [file 701_2023_5797_MOESM1_ESM.docx]
